# Time varying characteristic in somatosensory evoked potentials as a biomarker of spinal cord ischemic-reperfusion injury in rat

**DOI:** 10.3389/fnins.2024.1411016

**Published:** 2024-09-09

**Authors:** Kai Li, Jianwei Yang, Huaibo Wang, Xuejing Chang, Guanjun Liu, Ruiyang Xue, Weitao Guo, Yong Hu

**Affiliations:** ^1^Department of Spine Surgery, The Second Hospital Affiliated to Guangdong Medical University, Zhanjiang, Guangdong, China; ^2^Department of Orthopedics and Traumatology, The University of Hong Kong, Hong Kong, Hong Kong SAR, China

**Keywords:** spinal cord ischemia–reperfusion injury, somatosensory evoked potential, time-varying characteristic, biomarker, intraoperative monitoring, rat model

## Abstract

Spinal cord ischemic-reperfusion injury (SCIRI) could occurs during surgical procedures without detection, presenting a complex course and an unfavorable prognosis. This may lead to postoperative sensory or motor dysfunction in areas innervated by the spinal cord, and in some cases, permanent paralysis. Timely detection of SCIRI and immediate waring can help surgeons implement remedial intervention to prevent irreversible spinal cord injury. Therefore, it is crucial to develop a precise and effective method for early detection of SCIRI. This study utilized rat models to simulate intraoperative SCIRI and employed somatosensory evoked potentials (SEP) for continuous monitoring during surgery. In this study, SEP signal changes were examined in six groups with varying severities of SCIRI and one normal control group. SEP signal changes were examined during operations in different groups and correlated with postoperative behavioral and histopathological data. The result demonstrated specific changes in SEP signals during SCIRI, termed as time-varying characteristics, which are associated with the duration of ischemia and subsequent reperfusion. Time-varying characteristics in SEP could potentially serve as a new biomarker for the intraoperative detection of SCIRI. This finding is significant for clinical surgeons to identify and guide early intervention of SCIRI timely. Additionally, this measurement is easily translatable to clinical application.

## 1 Introduction

Spinal cord ischemic-reperfusion injury (SCIRI) refers to the exacerbated damage to the spinal cord structure and neural function caused by blood reperfusion after a period of ischemia in spinal cord tissue (Vidal et al., 2017; Hasan et al., 2022; Algahtani et al., 2022). This complication is not rare in clinical, primarily occurring during major thoracic and abdominal vascular surgeries, as well as spinal surgeries (Kato et al., 2015; Mathkour et al., 2020; Gerardi et al., 2022).

SCIRI is a complex process involving multiple cellular and molecular-level changes. It encompasses mechanisms such as oxidative stress, inflammatory response, vascular damage, cellular apoptosis, and necrosis, which collectively lead to tissue damage and functional impairment in the spinal cord (Na et al., 2019; Han et al., 2022; Rong et al., 2022). Therefore, several remedial interventions have been proposed to alleviate SCIRI in clinical practice (Behem et al., 2022; Mukai et al., 2022; Hou et al., 2022; Zheng et al., 2023; Chen et al., 2023). However, due to the irreversible nature of spinal nerve damage, these treatments are only effective when SCIRI is detected at an early stage. For patients who do not receive a timely detection, the lack of immediate treatment can lead to sensory or motor dysfunction in areas innervated by the spinal cord, or even permanent paralysis (Huang et al., 2022; Guo et al., 2023). Therefore, it is crucial to develop an intraoperative monitoring method capable of promptly detecting the occurrence of SCIRI.

Intraoperative monitoring using somatosensory-evoked potentials (SEP) has become an indispensable tool in surgical procedures, providing real-time monitoring of spinal cord function and potential spinal cord injury during operation procedure (Hu et al., 2001; Hu et al., 2003; Hattori et al., 2019; Park et al., 2021). However, SEP monitoring usually assesses the overall integrity of spinal cord neural function and does not clearly indicate the specific causes of potential injury. Recent research has confirmed the feasibility of using SEP for indicating localization in spinal cord injuries caused by various mechanical forces. In spinal cord injuries resulting from different mechanical mechanisms, SEP parameters exhibit distinct distribution patterns, suggesting a new method for rapidly identifying the causes of mechanical spinal cord injuries intraoperatively (Li et al., 2021; Li et al., 2023). This new measurement method also inspired our research to apply SEP for the detection of SCIRI.

SCIRI is a serious complication following surgical procedures, which is a great challenge in clinical practice because of its unpredictability, delayed onset, and severity. If SEP monitoring can detect the occurrence of intraoperative SCIRI and promptly alert the surgeon to take the appropriate intervention, it could effectively prevent postoperative neurological dysfunction. This study utilizes a rat model of SCIRI to explore this possibility. Our objective is to develop a SEP measurement as a biomarker that can accurately detect the occurrence of SCIRI, thereby providing crucial assistance in preventing and timely treating SCIRI during surgical procedures.

## 2 Materials and methods

### 2.1 Animals and study groups

All procedures were conducted in accordance with the guidelines of the Care and Use of Laboratory Animals (Institute of Laboratory Animal Resources, National Research Council, 1996). The study received approval from the Ethics Review Committee of Guangdong Medical University (GDY2302124) on March 1, 2023. Forty-two male Sprague–Dawley rats (specific-pathogen-free level), aged 8 to 9 weeks and weighing 280 to 320 g, were obtained (Liaoning Changsheng Biotech Co., Ltd., license No. SCXK (liao) 2020–0001). The rats were housed under suitable environmental conditions (temperature 20–25°C) and provided with food and water *ad libitum*.

**Figure 1:**
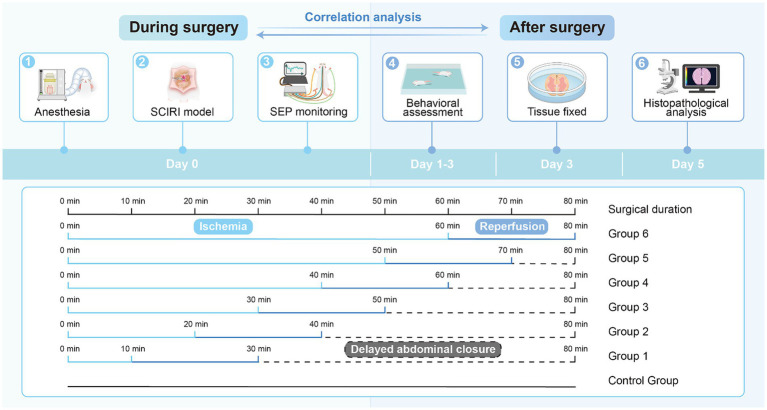
The timeline for the SCIRI model and the detection methods.

Forty-two rats were randomly divided into seven groups: one normal control group and six experimental groups (10-min ischemia group, 20-min group, 30-min group, 40-min group, 50-min group, and 60-min group, with reperfusion time consistent at 20 min for each group), with six rats in each group (Figure 1).

### 2.2 Establishment of animal model

To induce SCIRI, all rats were anesthetized using isoflurane (Zuoba, Guangzhou, China) and administered via a mask connected to an anesthesia machine (Ruivode, Shenzhen, China). Once successfully anesthetized, the rats were placed on a warm pad and their limbs were secured. The surgical area was cleansed and sterilized, and electrodes for SEP recording were applied. The abdominal aorta and left renal artery were exposed. The abdominal aorta was clamped at the level of the left renal artery, and reperfusion occurred upon releasing the arterial clamp, established the SCIRI rat model (Gokce et al., 2016; Zhao et al., 2019; Li et al., 2020). Each group had different ischemia durations (10–60 min) while maintaining the same reperfusion time (20 min). We standardized the duration of abdominal closure for each group of rats to 80 min. For instance, in the rats subjected to 10 min of ischemia, after 20 min of reperfusion, we did not immediately close the abdomen but waited for 50 min instead. This ensured consistency in the duration of surgical manipulation between the rats subjected to 10 min of ischemia and those subjected to 60 min of ischemia. The objective was to minimize bias and eliminate the influence of variations in surgical procedure duration. Subsequently, the rats were returned to their cages with unrestricted access to food and water.

### 2.3 Measurement of somatosensory evoked potentials

SEP was continuously measured during the surgery using a neural electrophysiology test system (Yirui Technology Co., Ltd., Zhuhai, China). To elicit SEP in the cranial sensory cortex, stimulating electrodes were inserted into the toes and tibialis anterior muscles of both lower limbs, applying a constant current stimulation to the tibial nerve at a frequency of 5.3 Hz and a pulse duration of 0.2 ms. The filtering range was set at 30-2000 Hz, and the signal was averaged over 300 trials. SEP latency and amplitude were recorded for each experimental group at normal values (baseline), at the ischemic time point (Ocl values), and at 5, 10, 15, and 20 min after reperfusion.

### 2.4 Behavioral evaluation

After completing the experiments, each group of rats was returned to their cages with unrestricted access to food and water, allowing for free movement. Behavioral assessments of the control group and the six experimental groups were conducted using the Basso, Beattie, and Bresnahan (BBB) rating scale on postoperative days 1, 2, and 3 (Basso et al., 1995). The rats were placed in an open field, and their scores ranged from 0 to 21, with 0 indicating no motor function and 21 indicating normal function. The hind limb function of the rats during ambulation was evaluated and recorded by two independent observers.

### 2.5 Tissue processing

After completing the behavioral assessments, the rats in the control group and the six experimental groups were euthanized by intraperitoneal injection of sodium pentobarbital. (Sigma, St. Louis, MO, USA). Cardiac perfusion was then conducted using 250 mL of 0.9% physiological saline and 250 mL of 4% paraformaldehyde. The entire lumbar spinal segment was extracted and immersed in a 4% paraformaldehyde solution. After 2 days of immersion, the spinal cord segments from the L3-L5 region were carefully and completely dissected from the vertebral canal. Each spinal cord specimen was then assigned a unique identification number, embedded in paraffin, and processed using a paraffin microtome (catalog number: RM2016, manufacturer: Leica) to obtain transverse sections with a thickness of 4 μm.

### 2.6 Histological staining

Histopathological examination of spinal cord tissue can more intuitively reveal the severity of spinal cord injury under a microscope. On day 3 after SCIRI, the severity of the spinal cord injury was comprehensively assessed by analyzing the expression levels of neurons, Nissl bodies within the specified region.

### 2.7 Hematoxylin and eosin (HE) staining

Sections were dewaxed with xylene, rehydrated in ethanol at varying concentrations, stained with hematoxylin (Servicebio, Wuhan, China), differentiated in 1% aqueous HCl, and then counterstained with eosin (Servicebio, Wuhan, China). Sections were dehydrated in ethanol and xylene and coverslipped with mounting medium.

### 2.8 Nissl staining

Sections were dewaxed with xylene, rehydrated in ethanol at varying concentrations, and then stained with Nissl staining solution (Boster, Wuhan, China) at 60°C for 40 min. Sections were dehydrated in ethanol and xylene and coverslipped with mounting medium.

### 2.9 Image acquisition and analysis

Utilizing an optical microscope, we acquired images of HE staining and Nissl staining (catalog number: HS6, manufacturer: Leica). Subsequently, we performed quantitative analysis of the spinal cord gray matter anterior horn region for HE staining and Nissl staining using ImageJ 1.47v (National Institutes of Health, USA).

### 2.10 Statistical analysis

Data analysis was performed using GraphPad Prism v9 software (GraphPad, USA).^1^ Initially, the Shapiro–Wilk test was employed to assess the normality distribution of these groups. The SEP data were presented as percentages, while the remaining data were expressed as mean ± standard error of the mean. The statistical significance was calculated by one-way ANOVA with a Tukey’s multiple comparison. Since SEP data were non-normally distributed, Spearman’s correlation analysis was used to examine the relationships between SEP and behavioral data, as well as SEP and histopathological data. A *p*-value of <0.05 was considered statistically significant.

## 3 Results

### 3.1 General situation

All rats successfully underwent SCIRI experiment without any intraoperative or post-anesthesia deaths. They regained consciousness within 2 h after anesthesia and were able to eat freely within 12 h. None of the experimental animals exhibited signs of incision infection or suture dehiscence.

### 3.2 The characteristic alterations of SEP in different severity of SCIRI

After the rat model was established, the SEP latency and amplitude were measured for each group. The results revealed that, with varying durations of ischemia and reperfusion, both the latency and amplitude of SEPs underwent changes. These alterations demonstrated a distinct Time-varying characteristic, being closely associated with the time of spinal cord ischemia and reperfusion (Table 1).

**Table 1:** Intraoperative SEP monitoring of SCIRI latency and amplitude time-varying characteristics.

Changes of SEP latency (%) in rats with SCIRI						
Group	Ischemia 10 min	Ischemia 20 min	Ischemia 30 min	Ischemia 40 min	Ischemia 50 min	Ischemia 60 min
Baseline	0	0	0	0	0	0
Occlusion	29.81	46.62	57.76	62.07	79.50	88.29
Reperfusion 5 min	21.70	36.04	62.24	63.66	82.66	95.95
Reperfusion 10 min	19.82	46.81	68.32	74.47	96.02	113.06
Reperfusion 15 min	13.51	42.66	81.61	93.02	110.59	129.73
Reperfusion 20 min	6.76	43.56	119.44	122.45	133.41	156.46

Changes of SEP amplitude (%) in rats with SCIRI						
Group	Ischemia 10 min	Ischemia 20 min	Ischemia 30 min	Ischemia 40 min	Ischemia 50 min	Ischemia 60 min
Baseline	0	0	0	0	0	0
Occlusion	−41.14	−58.53	−61.95	−67.68	−67.10	−73.36
Reperfusion 5 min	−43.70	−55.01	−64.07	−69.46	−69.03	−74.71
Reperfusion 10 min	−36.59	−54.09	−65.94	−71.92	−71.77	−76.49
Reperfusion 15 min	−29.88	−51.11	−68.79	−76.11	−74.86	−78.18
Reperfusion 20 min	−21.73	−52.12	−72.59	−77.02	−77.89	−80.88

Calculation of the changes in the latency or amplitude period of the SEP is performed as follows: (Injured latency or amplitude parameter - Baseline latency or amplitude parameter)/Baseline latency or amplitude parameter × 100%. *n* = 6.

**Figure 2:**
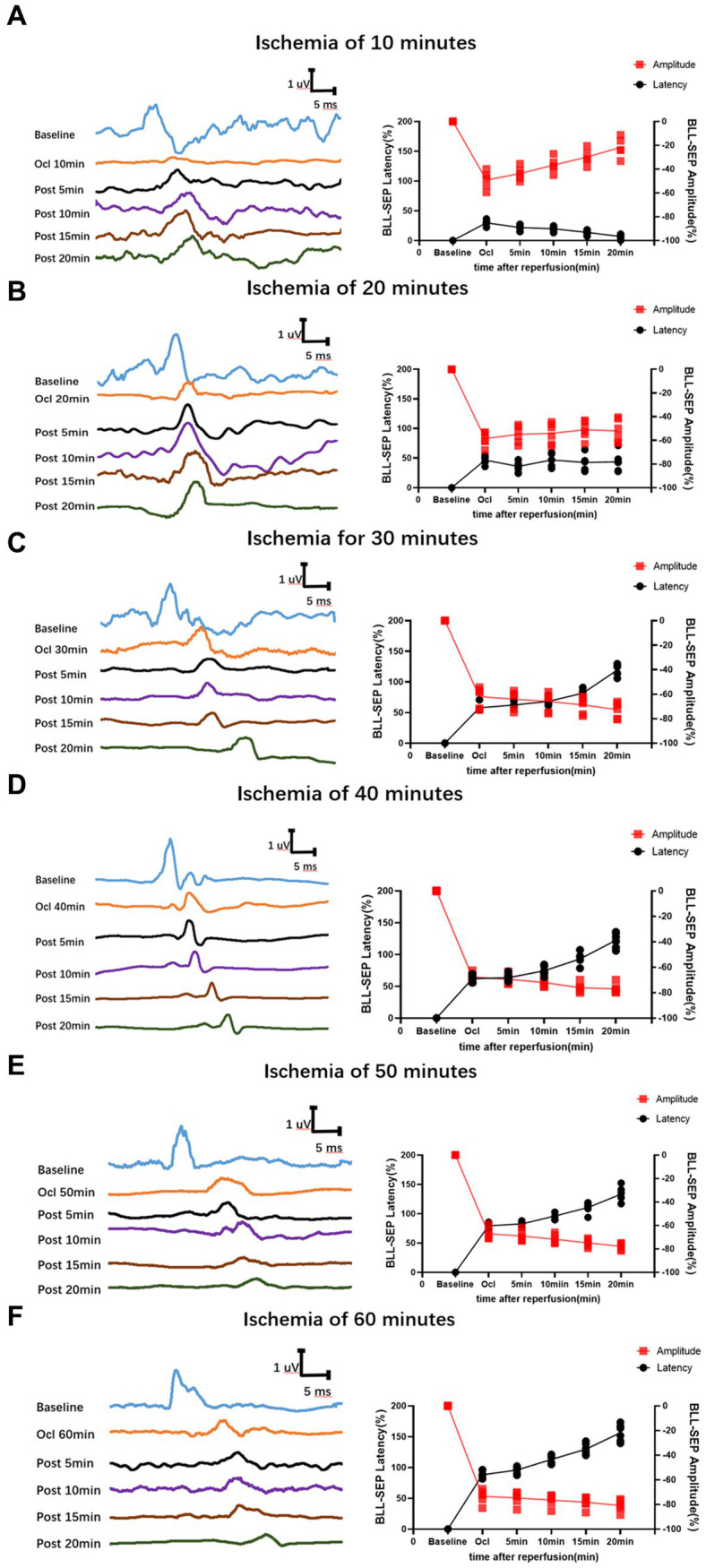
Time-varying SEP in waveform and latency/amplitude in rats with SCIRI. **(A)** 10-min ischemia group. **(B)** 20-min ischemia group. **(C)** 30 min ischemia groups. **(D)** 40 min ischemia groups. **(E)** 50 min ischemia groups. **(F)** 60 min ischemia groups. The reperfusion duration for each group is consistent at 5, 10, 15, and 20 min. Data were expressed as the percentage. (*n* = 6; BLL = Both lower limbs; Ocl = Occlusion).

In the group with 10 min of ischemia, SEP exhibited a mild extension in the latency of the ischemic time point (Ocl values) compared to the normal values (baseline). Additionally, there was a slight decrease in amplitude, indicating the onset of spinal cord conduction dysfunction at this at this moment. However, following 5, 10, 15, and 20 min of reperfusion after blood reperfusion, concurrent with the gradual restoration of spinal cord blood flow, SEP latency gradually decreased, and amplitude progressively increased. The SEP signals demonstrated a trend of gradual recovery (Figure 2A), Signifying a gradual recovery of neural conduction function.

In the group with 20 min of ischemia, the latency of the ischemic time point (Ocl values) exhibited an extended duration compared to the normal values (baseline), and the amplitude decreased, indicating ischemic damage to the spinal cord at this moment. Following 5, 10, 15, and 20 min of reperfusion after blood reperfusion, SEP values did not immediately exhibit improvement or exacerbation. Instead, they fluctuated around the values of the ischemic time point (Figure 2B), indicating that SCIRI injury did not occur at this time.

However, in the 30–60 min ischemia groups, as the ischemic duration extended, SEP exhibited a gradual prolongation of latency at the ischemic time point (Ocl value) and a gradual reduction in amplitude, indicating an increasing severity of spinal cord injury with prolonged ischemia. Subsequently, upon reperfusion of spinal cord blood, the electrophysiological signals of SEP showed a different trend compared to the first two groups. Following blood reperfusion at 5, 10, 15, and 20 min, the SEP latency gradually extends on the basis of the ischemic time point (Ocl value), and the amplitude decreases, suggesting a progressive worsening of spinal cord injury with prolonged reperfusion time (Figures 2C–F), indicating the occurrence of SCIRI during this time period.

### 3.3 Behavioral differences among different severity of SCIRI

**Figure 3:**
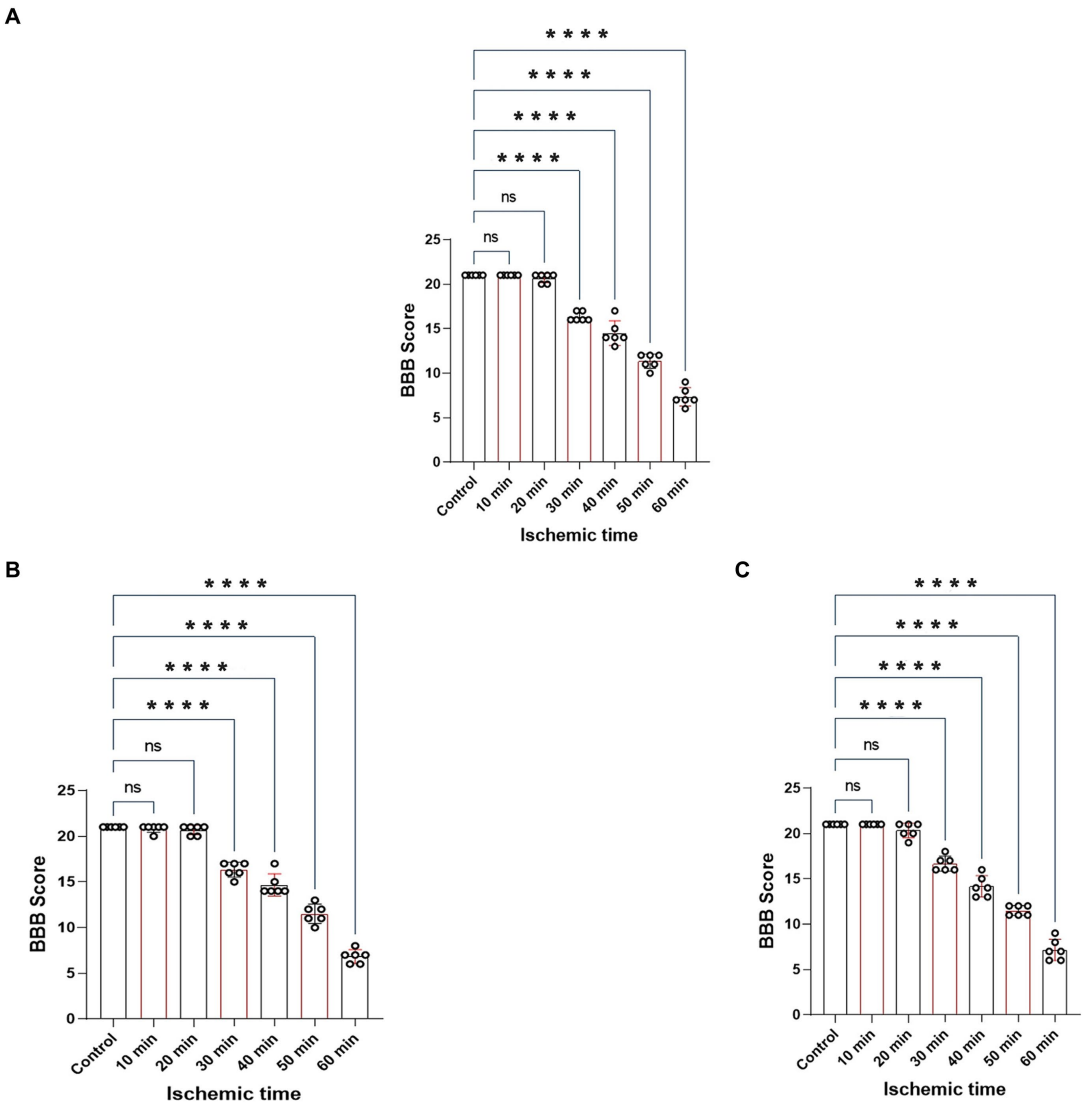
Represents the BBB scores of rats on day 1, 2 and 3 after SCIRI. **(A)** BBB score variations on day 1 after SCIRI. **(B)** BBB score changes on day 2 after SCIRI. **(C)** BBB score changes on day 3 after SCIRI. Data were presented as the mean ± SD. The statistical significance was calculated by one-way ANOVA with a Tukey’s multiple comparison (*n* = 6; ns: *p* > 0.05，indicating no significance; *p < 0.05, signifies that there was statistical significance; *p < 0.05, **p < 0.01, ***p < 0.001, ****p < 0.0001).

**Figure 4:**
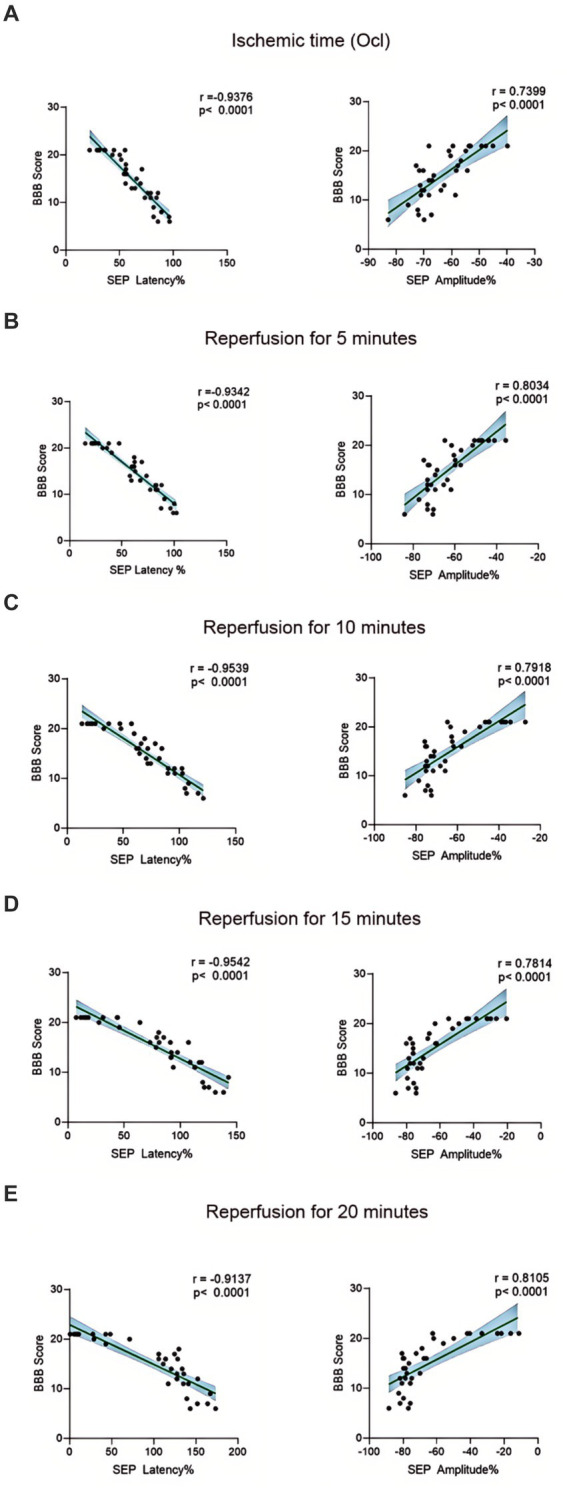
Scatterplots showing the relationship between SEP signal changes and behavioral outcomes correlation analysis in SCIRI. **(A)** Correlation analysis of SEP signal changes and behavior at different ischemia times. **(B–E)** Correlation analysis of SEP signal changes and behavior at 5, 10, 15, and 20 min of reperfusion at different ischemia times. The linear regression line of the best fit is represented by a solid line. The 95% confidence interval is represented by a dotted line, were conducted using Spearman’s correlation analysis (*n* = 6; Ocl = Occlusion).

The BBB scoring system stands as one of the most frequently utilized methodologies for behavioral assessment in the context of spinal cord injury in rats (Basso et al., 1995; Nagarajan et al., 2021). Using this method, we assessed hindlimb behavioral changes in different groups of rats on days 1, 2, and 3 after SCIRI after SCIRI. The results showed that the behavioral scores for the 10-min and 20-min ischemia groups were similar to those of the control group, with no statistically significant differences (*p* > 0.05). However, after 30 min or more of ischemia, the behavioral scores of the rats were significantly lower than those of the control group (*p* < 0.05). Additionally, as the ischemia duration increased, the hind limb behavioral scores progressively declined, indicating a gradual deterioration of neural function (Figures 3A–C). Subsequently, we performed a correlation analysis between the behavioral changes on day 3 and SEP signal variations in the rats. The results revealed a high correlation between the trends in SEP signal changes and the severity of behavioral impairments as the ischemia and reperfusion durations varied (Figures 4A–E), confirming consistency between the two sets of results.

### 3.4 HE staining differences among different severity of SCIRI

**Figure 5:**
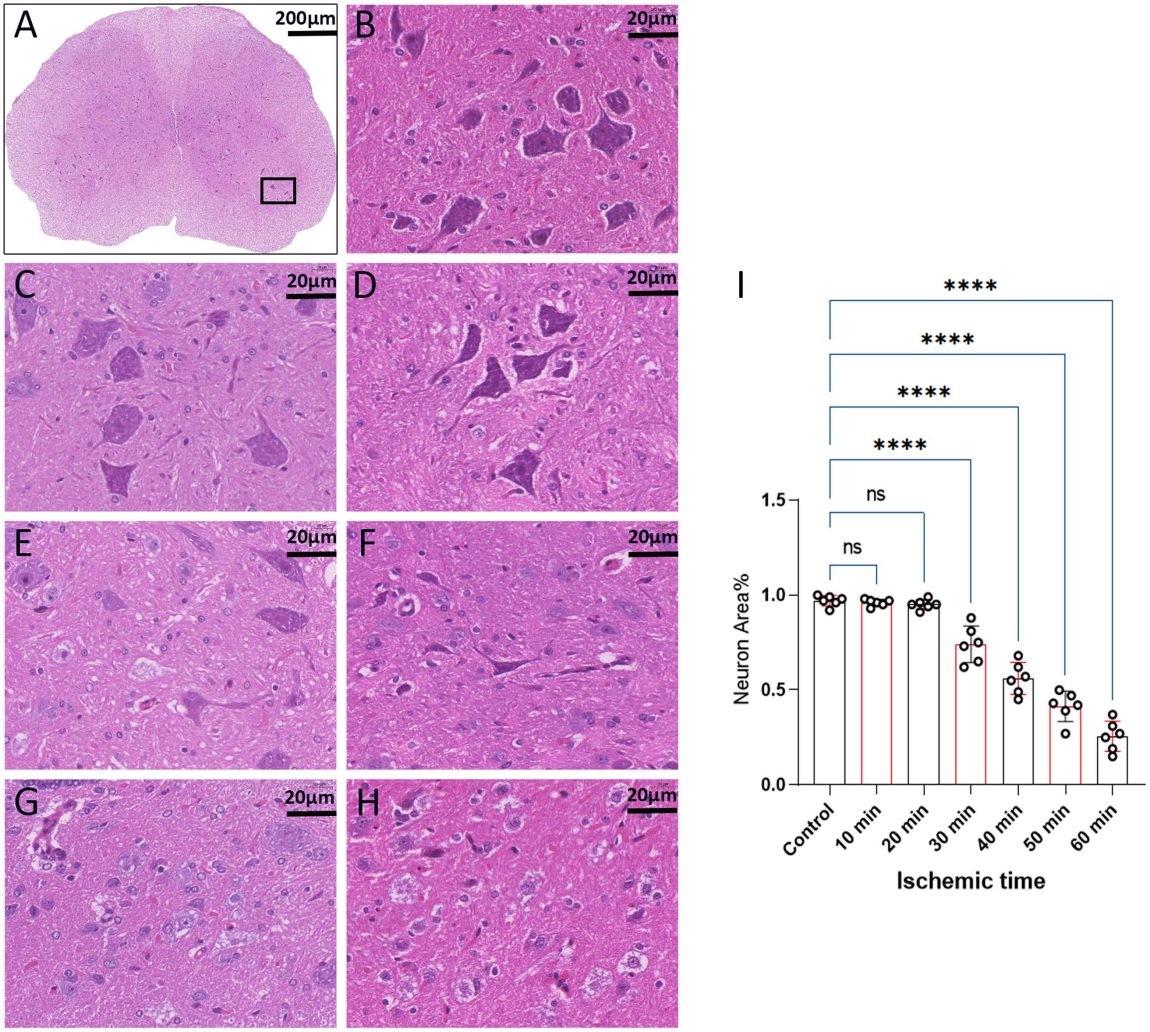
Spinal cord sections on day 3 after SCIRI (HE staining, light microscope). **(A)** Each image was selected from the anterior horn region of the spinal cord (×4, Scale bar: 200 μm). Boxes represent the magnified region. **(B)** Enumeration and morphology of motor neurons in this region for the control group (×40, scale: 20 μm). **(C–H)** Enumeration and morphology of motor neurons in this region for the 10 to 60 min ischemia groups (×40, scale: 20 μm). **(I)** Data were presented as the mean ± SD. The statistical significance was calculated by one-way ANOVA with a Tukey’s multiple comparison (*n* = 6; ns: *p* > 0.05，indicating no significance; *: *p* < 0.05, signifies that there was statistical significance; *p < 0.05, **p < 0.01, ***p < 0.001, ****p < 0.0001).

**Figure 6:**
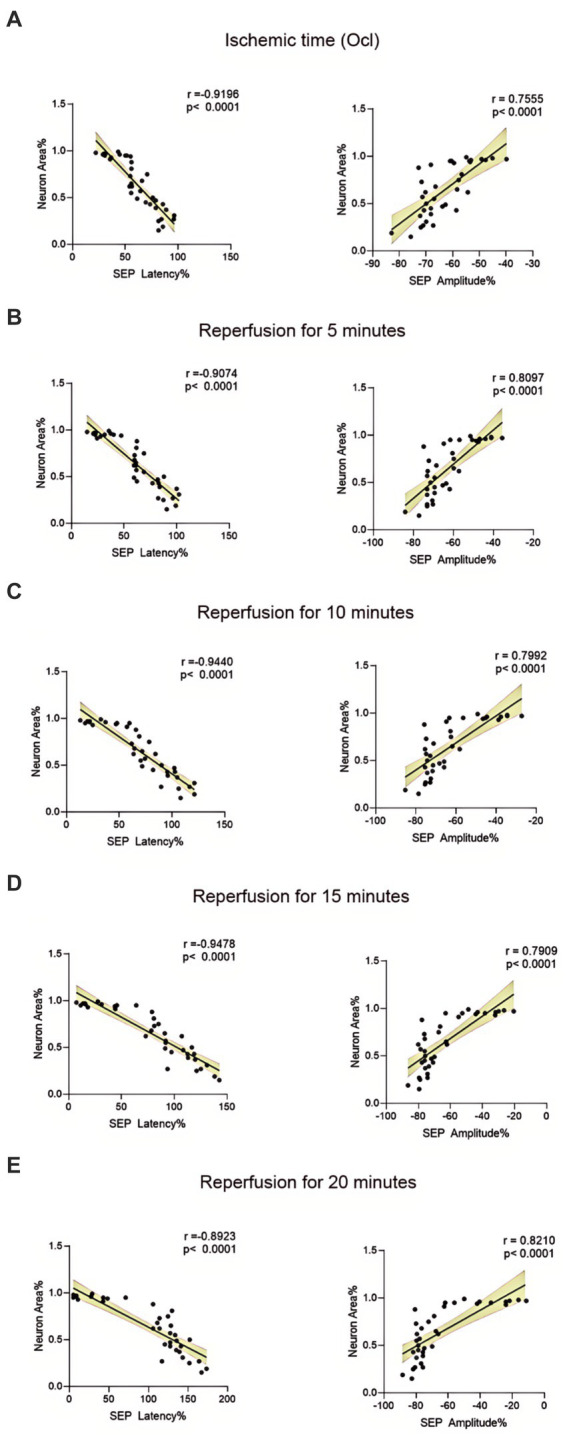
Scatterplots showing the relationship between SEP signal changes and HE staining outcomes correlation analysis in SCIRI. **(A)** Correlation analysis of SEP signal changes and HE staining at different ischemia times. **(B–E)** Correlation analysis of SEP signal changes with HE staining at 5, 10, 15, and 20 min of reperfusion at different ischemia times. The linear regression line of the best fit is represented by a solid line. The 95% confidence interval is represented by a dotted line, were conducted using Spearman’s correlation analysis (*n* = 6; Ocl = Occlusion).

The structural morphology of spinal anterior horn motor neurons in each group was assessed using HE staining within the same region (Figure 5A). In the 10-min and 20-min ischemia groups, the morphological structure of motor neurons in the anterior horn of the spinal cord appeared normal. The neuronal nuclei exhibited deep staining, maintained a complete circular shape, showed no signs of swelling, and displayed uniform cytoplasmic staining (Figures 5B–D), and compared to the control group, there was no statistically significant difference (*p* > 0.05). However, after 30 min or more of ischemia, there was a significant reduction in the number of neurons compared to the control group (*p* < 0.05). Additionally, as the ischemia duration increased, the neurons gradually atrophied, the cytoplasmic staining became lighter, the surrounding matrix disappeared, leading to vacuole formation, and the nuclei condensed. This indicates that with prolonged ischemia, the number of normal neurons gradually decreased (Figures 5E–H), indicating an increasing severity of spinal cord injury after SCIRI (Figure 5I). Subsequently, we conducted a correlation analysis between the pathological changes in neurons and SEP signal variations. The results revealed a high correlation between the trends in SEP signal changes and the degree of neuronal necrosis as the ischemia and reperfusion durations varied (Figures 6A–E), indicating consistency between the two sets of results.

### 3.5 Nissl staining differences among different severity of SCIRI

**Figure 7:**
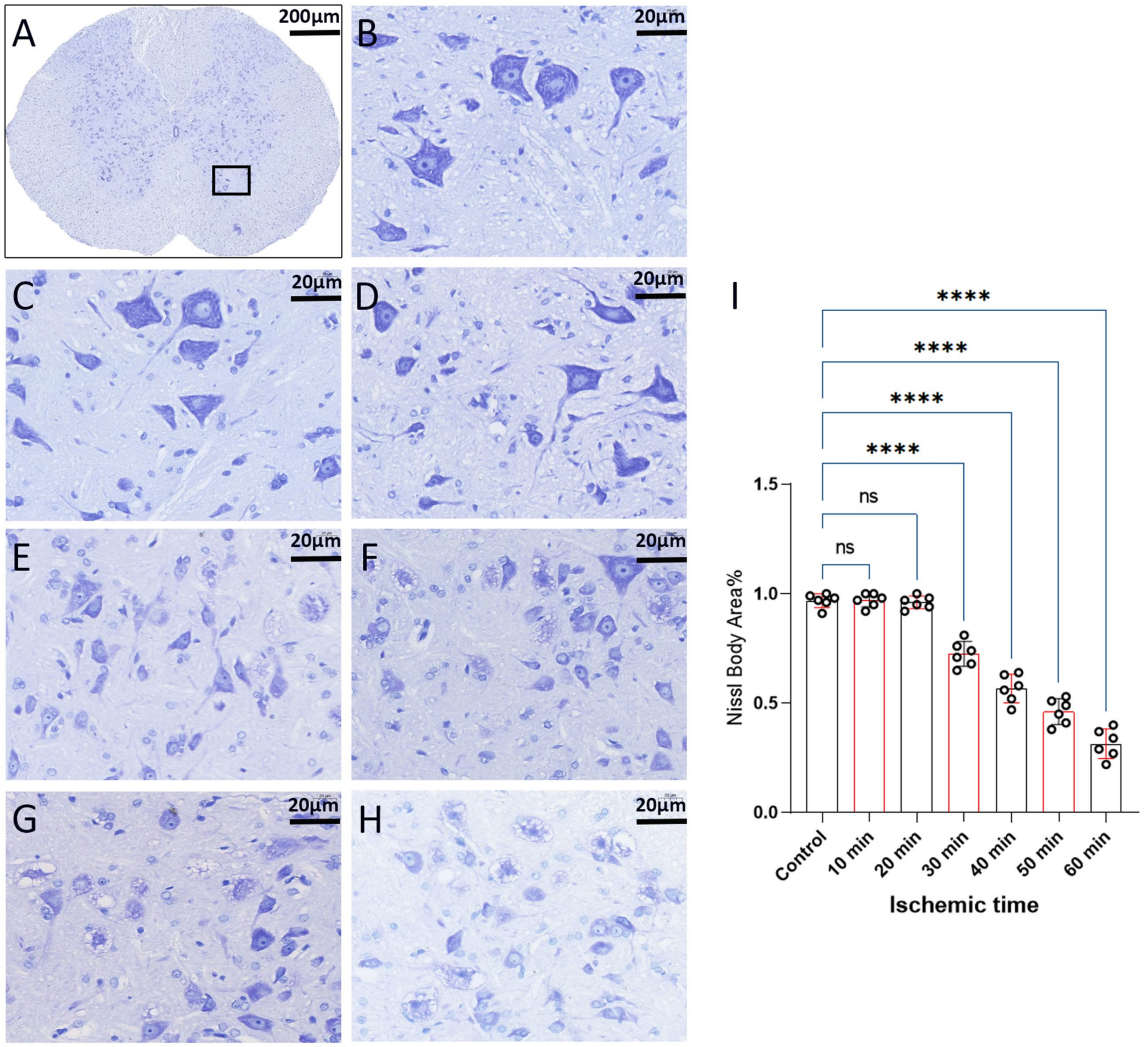
Spinal cord sections on day 3 after SCIRI (Nissl staining, light microscope). **(A)** Each image was selected from the anterior horn region of the spinal cord (×4, Scale bar: 200 μm). Boxes represent the magnified region. **(B)** Nissl body content in this region for the control group (×40, scale: 20 μm). **(C–H)** Nissl body content in this region for the 10 to 60 min ischemia groups (×40, scale: 20 μm). **(I)** Data were presented as the mean ± SD. The statistical significance was calculated by one-way ANOVA with a Tukey’s multiple comparison (*n* = 6; ns: *p* > 0.05，indicating no significance; *p < 0.05, signifies that there was statistical significance; *p < 0.05, **p < 0.01, ***p < 0.001, ****p < 0.0001).

**Figure 8:**
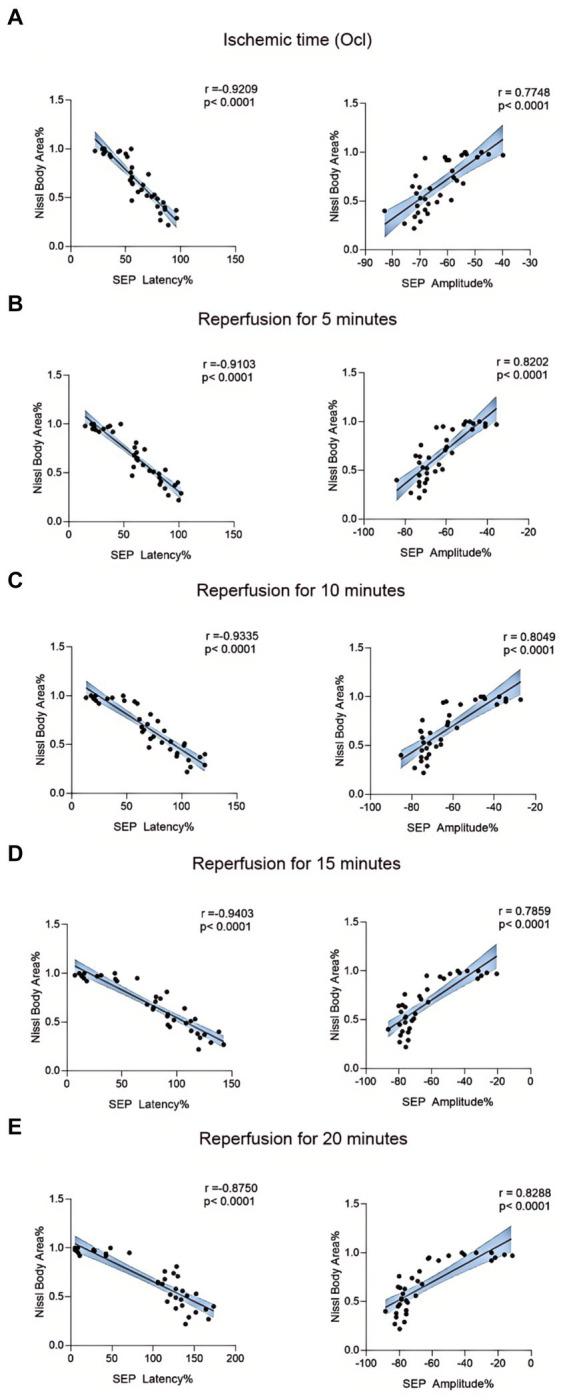
Scatterplots showing the relationship between SEP signal changes and Nissl staining outcomes correlation analysis in SCIRI. **(A)** Correlation analysis of SEP signal changes and Nissl staining at different ischemia times. **(B–E)** Correlation analysis of SEP signal changes with Nissl staining at 5, 10, 15, and 20 min of reperfusion at different ischemia times. The linear regression line of the best fit is represented by a solid line. The 95% confidence interval is represented by a dotted line, were conducted using Spearman’s correlation analysis (*n* = 6; Ocl = Occlusion).

We employed Nissl staining to observe the content of Nissl bodies in the same region of the spinal cord anterior horn in each group (Figure 7A). In the 10-min and 20-min ischemia groups, the morphology of motor neurons in the anterior horn of the spinal cord appeared normal, with a rich presence of Nissl bodies (Figures 7B–D), and compared to the control group, there was no statistically significant difference (*p* > 0.05). However, after 30 min or more of ischemia, the number of Nissl bodies decreased significantly compared to the control group (*p* < 0.05). Additionally, as the ischemia duration increased, the number of Nissl bodies in this region gradually decreased, and Nissl bodies progressively vacuolated (Figures 7E–H), suggesting an increasing severity of spinal cord injury after SCIRI (Figure 7I). Subsequently, we conducted a correlation analysis between the pathological changes in Nissl bodies and SEP signal variations. The results revealed a high correlation between the trends in SEP signal changes and the decreased number of Nissl bodies as the ischemia and reperfusion durations varied (Figures 8A–E), confirming consistency between the two sets of results.

## 4 Discussion

In clinical practice, the prevention and treatment of SCIRI remain a big challenge. Due to the inability to obtain tissue samples from clinical patients and the difficulty in making a definitive diagnosis, some published reports only describe “suspected” cases of SCIRI following surgery, lacking sufficient evidence to support the diagnosis (Kato et al., 2015; Antwi et al., 2018; Papaioannou et al., 2019; Acharya et al., 2021; Malinovic et al., 2021; Algahtani et al., 2022; Hasan et al., 2022). The lack of effective monitoring methods is the primary reason for the failure to achieve early detection and treatment of SCIRI. This experimental study examined the intraoperative monitoring value of SEP in detecting SCIRI. A new characteristic of SEP signal changes, referred to as the “time-varying characteristic,” as a novel biomarker that can specifically detect the occurrence of SCIRI. This will become an indicator for intraoperative neurophysiological monitoring, providing a novel scientific approach for the prevention, intervention, and early treatment of SCIRI.

Intraoperative SEP monitoring has become an essential tool used to assess the functional integrity of sensory pathways during surgical procedures (Hu et al., 2003; Ji et al., 2013; Li et al., 2023). The use of SEP helps to prevent the risk of spinal cord damage by providing real-time feedback to surgeons about the patient’s neurological status (Hu et al., 2003; Ji et al., 2013; Park et al., 2021; Guo et al., 2023). However, intraoperative SEP monitoring can only detect the integrity of neurological function, without detail message regarding injury mode and location (Cui et al., 2021; Li et al., 2023;). Currently, few studies elucidated the time-varying characteristics in SEP signals during the occurrence of SCIRI. Therefore, our study to explore and analyze SEP signal variations during SCIRI from a novel perspective to better understand these changes. Motor-evoked potentials (MEP) is another electrophysiological test to detect intraoperative injury to the spinal cord, which is suggested as the effective monitoring for SCIRI (Hattori et al., 2019; Li et al., 2023). However, MEP is easily disappeared when the injury happened. In SCIRI model, MEP showed too sensitive to the ischemia, so that it is not measurable during subsequent ischemia–reperfusion injury period (Li et al., 2023).

The clinical issues described above necessitate solutions derived from fundamental experimental research. In the last decade, there has been continuous exploration of SCIRI both domestically and internationally, accompanied by the refinement of animal models (Alizadeh et al., 2019). The primary approach involves inducing a rat model of SCIRI by occluding the aortic arch between the left carotid artery and the left subclavian artery (Dong Y. et al., 2023) or by clamping the abdominal aorta at the level of the left renal artery (Gokce et al., 2016; Zhao et al., 2019; Li et al., 2020). Both methods have been proven effective in inducing SCIRI. In this study, we employed the method of the abdominal aorta clamping at the level of the left renal artery to establish rat SCIRI models with varying ischemic durations. SCIRI was successfully induced in our study 30 min or more after spinal cord ischemia, aligning with previous research findings and confirming the adherence of our rat model to established standards. Intraoperative SEP monitoring was employed to dynamically observe the evolving characteristics of SEP signals during the SCIRI process. Correlation analyses were conducted between postoperative behavior and histopathological results with changes in SEP signals.

This study found that during a 10-min ischemic, following blood reperfusion. SEP potential signals demonstrated a trend of gradual recovery. This implies that brief periods of spinal cord ischemia do not lead to the occurrence of SCIRI. In the case of ischemia for 20 min, SEP values did not immediately exhibit improvement or exacerbation after blood reperfusion. Instead, they fluctuated around the values of the ischemic time point (Ocl values). Nevertheless, postoperative behavior and histopathological did not show any unusual changes (*p* > 0.05). We hypothesize that this phenomenon may be attributed to ischemic spinal cord injury. Due to the short ischemic duration, no SCIRI occurred upon blood reperfusion. Postoperatively, rats in this experimental group did not exhibit behavioral anomalies or histopathological changes, indicating the reversibility of this short-term ischemic spinal cord injury. In future studies, extending the monitoring time could provide insights into the long-term changes in neuroelectrical signals with this pathological condition. However, in the 30–60 min ischemia groups, after reperfusion of spinal cord blood, the signals of SEP showed a different trend compared to the first two groups. Based on the ischemia time point (Ocl value), the SEP latency gradually prolonged, and the amplitude gradually decreased, indicating a progressive deterioration in spinal cord conduction function, suggesting the occurrence of SCIRI during this time period. Moreover, the severity of spinal cord injury worsened with prolonged ischemia and reperfusion time. Postoperative behavioral and histopathological assessments similarly confirmed the monitoring results of SEP, and correlation analysis demonstrated consistency among the three outcomes. Through further extraction and analysis of SEP signals, we discovered unique alterations in SEP during SCIRI. Specifically, when SCIRI occurs, it produces specific variations depending on the duration of spinal cord ischemia and reperfusion. Importantly, these changes do not manifest in normal conditions or other types of spinal cord injuries. We term this phenomenon “the time-varying characteristic of SEP.” These time-varying characteristic can dynamically reflect the severity of SCIRI. This discovery facilitates surgeons in identifying the occurrence of SCIRI during the surgical process and possesses a distinct pioneering significance.

**Figure 9:**
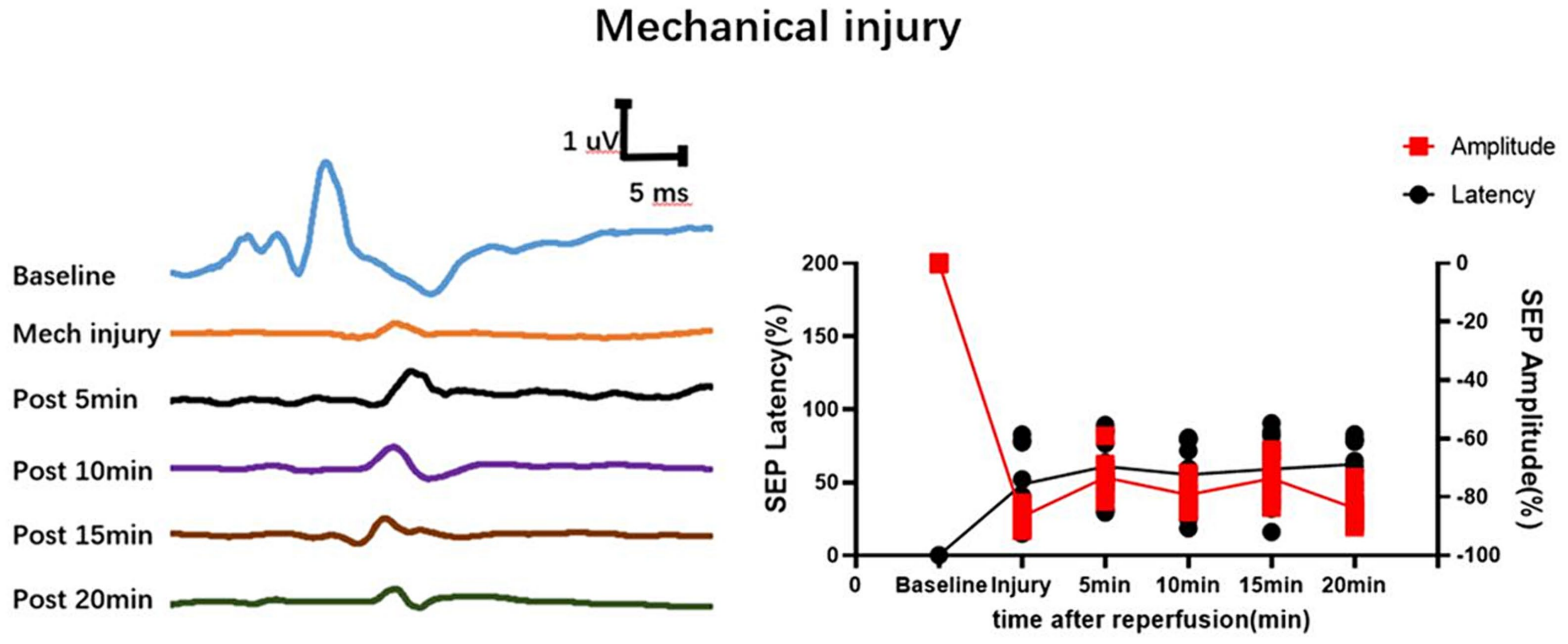
Depicts the waveform changes of SEP in mechanical spinal cord injury. Mechanical spinal cord injury (such as contusion, displacement, and traction) differs from SCIRI in that its SEP waveform remains fixed within a short period of time and does not exhibit time-varying characteristics. Data were expressed as the percentage (*n* = 10).

The time-frequency analysis of SEP showed paramount significance in spinal cord research (Hu et al., 2001; Hu et al., 2003; Hattori et al., 2019; Cui et al., 2021). It elucidates the functional status of the spinal cord, facilitates the detection and localization of spinal cord injuries, monitors therapeutic efficacy and rehabilitation processes, and delves into the pathophysiological mechanisms underlying spinal cord disorders (Ji et al., 2013; Cui et al., 2021; Li et al., 2021). This methodology furnishes a wealth of information and robust tools, thereby propelling advancements in spinal cord research and clinical applications. In a previous experimental study, SEP signal changes were investigated in different mechanical spinal cord injury models (such as contusion, displacement, and traction) (Li et al., 2023). In various mechanical spinal cord injuries, SEP parameters show different distribution patterns, leading to a new method for rapid intraoperative identification of the cause of spinal cord injury (Li et al., 2023). We further conducted an analysis of the SEP datasets obtained from previous experimental studies on mechanical spinal cord injurie. In the context of mechanical spinal cord injury, the latency of SEP prolongs, and the amplitude diminishes post-injury. Over a brief duration, SEP fluctuates around the injury threshold without exhibiting time-varying characteristic. However, during the occurrence of SCIRI, SEP undergoes signal alterations based on the injury threshold (Ocl value). These signal changes correlate with both ischemic and reperfusion times, showcasing distinctive time-varying characteristic (Figures 2C–F). In this study, SEP monitoring was used to investigate SCIRI induced by ischemia and revealed distinct characteristics compared to mechanical spinal cord injury (Figure 9). This demonstrated the potential use of the unique time-varying characteristics of SEP changes as a biomarker for definite detection of SCIRI during surgery.

The significance of this study lies in the unpredictability and severity of SCIRI in clinical practice. This phenomenon frequently occurs during surgical procedures, and due to the absence of effective intraoperative detection methods, the occurrence of SCIRI is often overlooked, leading to serious clinical consequences in postoperative patients. Presently, SCIRI poses a significant challenge in spinal surgery (Huang et al., 2023). If we can detect the occurrence of this phenomenon during surgery, early and effective intervention measures can be implemented to prevent irreversible spinal cord damage (Dong X. et al., 2023). Addressing these clinical needs, we utilized continuous intraoperative SEP monitoring to observe changes in electrophysiological signals during SCIRI in rats. Building upon this, we discovered a specific change in SEP during the monitoring of spinal cord ischemia–reperfusion injury, termed “time-varying characteristic.” This time-varying characteristic could potentially serve as novel intraoperative biomarkers for the definitive detection of SCIRI. Simultaneously, SEP has emerged as the primary method for monitoring spinal cord function during surgical procedures, gaining widespread recognition in clinical practice (Hu et al., 2001; Hu et al., 2003; Park et al., 2021). Therefore, the results of this study can be easily translated into clinical applications (Cui et al., 2021).

There are limitations to this study. Further validation of the time-varying characteristic of SEP is needed through more animal experiments. Its application in clinical surgeries also requires verification to establish its reliability as a detection criterion. Additionally, the timing and strategies for repair of neurologic injury in SCIRI need further investigation.

In summary, this study firstly introduce time-varying analysis of SEP to detect SCIRI in animal model. It provides evidence of the genuine occurrence of SCIRI, and demonstrates the capability of SEP to detect the onset of SCIRI during surgery. Furthermore, the occurrence of SCIRI is closely linked to the duration of spinal cord ischemia. Notably, when SCIRI occurs, SEP exhibits a prominent time-varying characteristic, suggesting its potential as a biomarker for definite detection of SCIRI during surgery.

## Data Availability

The raw data supporting the conclusions of this article will be made available by the authors, without undue reservation.

## Footnotes

^1^ www.graphpad.com
